# Supplementary material for: False memory in a second language: The importance of controlling the knowledge of word meaning
Source: PLoS One. 2023 May 11;18(5):e0285747. doi: 10.1371/journal.pone.0285747 (PMC10174556; doi:10.1371/journal.pone.0285747)
Supplement: Attachment — Submitted filename: Response to Reviewers.pdf [file pone.0285747.s001.pdf]

Response to Reviewers

[PONE-D-22-27134]

ACADEMIC EDITOR (Margarida Vaz Garrido)

We would like to express our sincere thanks for your carefully review and comments on our submission. In response, we have undertaken an extensive revision of the manuscript.

EDITOR COMMENT: 1. While replication studies are relevant, and the authors even identified and addressed some methodological issues from Beato and Ardnt's study, I concur with Reviewer 1 regarding the lack of theoretical advance or novel contribution. In the revised version, the authors should emphasize in the introduction and the discussion how this replication and the methodological issues you are addressing might contribute to informing and advancing theory.

> **RESPONSE:** Following your feedback and Reviewer's 1 suggestions, we have included more information both in the introduction (see page 8) and the discussion sections (see pages 27-29 and 33-34) to emphasize how our experiment contribute to advancing the theory.

EDITOR COMMENT: 2. Reviewer 2 suggests addressing the fuzzy-trace theory. I suggest at least some mention of explanations about L1_L2 processing differences (e.g., cognitive, affective) that might apply to these results.

> **RESPONSE:** As the Editor and Reviewer 2 suggest, we have mentioned the fuzzy-trace theory (FTT) on page 4. We do not discuss this theory further in the paper because the study does not aim to disentangle whether the AMF or the FTT account better for the results; thus, our manipulations were not designed to allow us to do so. In fact, in the DRM literature, gist extraction and associative activation processes are commonly confounded: lists usually have a solid thematic consistency and studied words are usually strongly associated with the critical lure, which is associated with gist extraction and activation processes, respectively. We discussed the results in terms of associative processes in order to bridge language and memory literature and allow an integrated discussion of the results.

EDITOR COMMENT: 3. Some methodological choices should also be justified. Why did the authors use a between design instead of comparing L1 and L2 performance within the same participants (Reviewer 2)? Why did the authors use a self-reported measure of L2 proficiency instead of a standardized test (Reviewer 1)? Is the self-reported score significantly above average? Ideally, given that performance in L2 might be related to several other variables, all participants should pass the test and then be randomly assigned to one of the conditions.

> **RESPONSE:** First, as Reviewer 2 suggested, we have included the explanation of why we did not test participants in both languages in the Procedure section (see page 15).
>
> Second, addressing Reviewer's 1 concern, we have made the reader aware of this limitation of a self-reported measure of L2 proficiency and we have suggested the use of other standardized

questionnaires in future research (see page 32-33). Although not having a standardized measure of L2 proficiency is a limitation, we confirmed that our subjective (i.e., self-report) and objective (i.e., translation test score) L2-proficiency measures were highly and positively correlated ($r = .74$, $p < .001$). Furthermore, the correlation analyses that we run confirmed that the L2-proficiency measure used in this experiment, even though it was self-reported, provides interesting and useful data to test the theories.

EDITOR COMMENT: 4. While (as the authors did) it is important to control for knowledge of the words' meaning (and repeating the analysis including only the words that were known), it would be important to examine whether L2 proficiency (even if self-reported) moderates the results (Reviewer 3). It would be expected that the higher the proficiency, the closer the performance to the L1 condition? This analysis could also be implemented using the "translation" indicators.

RESPONSE: Thank you for this suggestion. We agree with the Editor and with Reviewer 3 that it is important to examine whether L2 proficiency moderates our results. For that aim, in the new version of the manuscript we have run some correlation analyses that raised interesting results. We have included these results in pages 25-26 (see algo Table 5) and discussed them in page 30.

[Pages 25-26] "In addition, it would be important to examine whether the self-reported L2 proficiency moderates the BAS effect on L2 false memory. Even though to examine the effect of L2 proficiency was not among the main goals of this work, some analyses have been run to test whether the higher the L2 proficiency, the closer the performance to the L1 condition, namely, the higher the false recognition. This would be the expected result based on the RHM as it states that when L2 speakers become more proficient, the conceptual links from L2 words to the concepts become stronger. As shown in Table 5, Pearson's correlations indicated that L2-proficiency was not related to the true recognition of the studied words, but it was positively correlated with the false recognition of the critical lures both from high-BAS and low-BAS DRM lists. That is, the more proficient the participants were in their L2, the greater the false recognition rates of L2 critical lures from both high- and low-BAS lists. Moreover, the results were the same when only the L2 words whose meaning was known were included in the correlation analyses. In other words, irrespective of the knowledge of L2-word meaning, L2-proficiency seem to play an important role in the reported BAS effect on false recognition in the L2."

[Page 30] "Moreover, our data also showed evidence that the self-reported L2 proficiency plays an important role in the L2 false memory effect. Specifically, we found that the higher the L2 proficiency, the greater the false recognition of L2 critical lures from high-BAS and low-BAS lists. These results are consistent with previous studied [39,49] and support the predictions of both the RHM and the AMF. The RHM predicts that once L2 speakers become more proficient, their conceptual links from L2 lexical representations strengthened, activating the conceptual representation from L2 words faster and more directly as their proficiency in this language increases. Following the AMF, this predicts a greater production of false memories as L2 proficiency improves."

EDITOR COMMENT: 5. As long as there is an a priori hypothesis regarding the specific comparison, I have no problems with the post-hoc test following a non-significant interaction (Reviewer 3). Still, this needs to be better justified.

> **RESPONSE:** Thank you for noticing that these post hoc analyses were not well justified. We have included a better explanation about why we conducted this post hoc test after a marginally significant interaction, specifying our a priori hypothesis (see page 25).
>
> > "Finally, there was a marginally significant interaction Word type x BAS, $F(1, 118) = 3.83$, $p = .053$, $\eta^2_p = 0.031$. As we had an a priori hypothesis regarding this interaction (i.e., BAS will have an effect in false recognition, but not in true recognition), we continue with the analysis of this marginally significant interaction."

REVIEWER #1

We thank Reviewer 1 for taking time to give feedback on our manuscript. All the thoughtful comments on our initial submission have been taken into account in the manuscript. We believe that it is now much improved and that it will make a much stronger contribution to the literature.

REVIEWER COMMENT: Despite the replication of a single study with the implementation of methodological improvements is always desirable and welcome, the current work lacks of any novelty either by the inclusion of a new variable or a follow-up study extending the mere replication of the results. Therefore, there is a lack of theoretical advance or novel contribution.

> **RESPONSE:** We believe that our work is contributing to advancing the theory and, thus, add a novel contribution to the scientific literature. We have emphasized both in the introduction (see page 8) and the discussion (see pages 27-29 and 33-34) how this replication and the methodological issues we are addressing represents an important theoretical advance.
>
> > [Page 8] "Furthermore, why is it important to conduct this replication study and to address the methodological limitations of the previous article [49]? On the one hand, due to the replication crisis that the field of psychology has been suffering [57], and considering that reproducibility should be one of the core principles of science, replication studies are a way to improve this matter. Following the Open Science Collaboration [58], a single study provides tentative evidence, while a successful replication provides confirmatory evidence. On the other hand, addressing the methodological limitations will help us clarify and validate Beato and Arndt's findings on the effect of BAS on false memories in the L2 and this will have theoretical implications. The previously found higher false recognition in the L1 than L2, and the fact that the BAS effect occur not only in the L1 but also in the L2, seem to go in line with the predictions of the RHM and the AMF, as it was explained above. However, a core assumption of the predictions of these two theoretical explanations is that participants know the meaning of the words, otherwise they will not be able to access the conceptual store from the lexical representations and no activation would be spread to associated concepts. Therefore, as Beato and Arndt [49] provided evidence that second-language learners' knowledge of L2 stimuli was far from perfect, there is an alternative explanation for these

memory effects. Precisely, a plausible explanation would be that differences in the knowledge of L2-word meaning across languages can explain some or all language dominance effects on false memory. In the present experiment we will include a measure of the knowledge of L2-word meaning to rule out this plausible interpretation of previous findings and contribute advancing the theory."

[Page 27] "This approach is justified given the limited reproducibility of research in the field of psychology [57] and because it helped us clarify and validate Beato and Arndt's [49] findings on the effect of BAS on false memories in the L2. Below, while we discuss the main results, we will highlight the theoretical implications and the novel contributions of the present work."

[Page 28] "The most significant improvement and novel contribution raised by our experiment over the prior work by Beato and Arndt [49] was to control the knowledge of L2-word meaning. This was necessary since Beato and Arndt reported that participants had far from perfect knowledge of L2 stimuli and the knowledge of word meaning in their study differed between the high-BAS and low-BAS lists, making it difficult to interpret the BAS effect on false recognition in the L2. To control for the knowledge of L2-word meaning, first, we conducted a previous study to obtain an estimate of the knowledge that L1 speakers (i.e., Spanish) had for the meaning of L2 words (i.e., English). This previous study was useful when building the L2 DRM lists because it allowed us: 1) to include words that were known by most of the participants (participants had near-perfect knowledge of the critical lures, which is vital in order to observe the false memory effect); and 2) to ensure that the knowledge of L2-word meaning was control between high- and low-BAS lists. Second, at the end of the experiment, we asked participants to conduct a translation test, so we had an objective measure of their knowledge regarding the meaning of L2 words. These two approaches provided us with key data to evaluate the effect of BAS in L1 and L2 false recognition ruling out the possibility that the knowledge of L2-word meaning could explain these false memory effects. […]"

[Page 29] "In addition, the ANOVAs performed only with the words whose meaning was known to the participants clarifies the possible effect that the lack of knowledge of the L2 words could have on our results and conclusions, providing the scientific literature with solid evidence that supports the RHM and the AMF."

[Page 33-34] "As a final remark, this study makes the following contributions to the scientific literature […]."

REVIEWER COMMENT: Here there are several comments the authors might consider in future submissions: In page 6, starting in line 124, three main issues are raised from Beato and Ardnt: a- the use of 3 lures instead of 1; b- DRM list varied in levels of BAS and FAS; c- Not control of participants' meaning knowledge for L2 words. In page 7, starting in line 146, authors presented what they implemented to overcome these three issues but in a different order (c, a, X, b) which disturb the readiness of the manuscript. In the discussion it is again found this lack of order. Including such detailed order of hypotheses and objectives in the introduction should have prompt to follow it in the discussion.

RESPONSE: Thank you for pointing out the lack of order in the presentation of the limitations of the previous study. We have changed the order in which those limitations are presented, both in the introduction and the discussion, so that they follow the same order throughout the document: a- Not control of participants' meaning knowledge for L2 words; b- the use of 3 lures instead of 1; c- DRM list varied in levels of BAS and FAS (see page 7 in the introduction and page 29 in the discussion).

REVIEWER COMMENT: Moreover, the presented study itself also presents some limitations that should have been the least mentioned there: for example, instead of self-report on second language command, an standardize test could have been used.

RESPONSE: We have made the reader aware of this limitation and we have suggested the use of other standardized questionnaires in future studies (see page 32-33). Although we agree with the reviewer that not having a standardized measure of L2 proficiency is a limitation, we confirmed that our subjective (i.e., self-report) and objective (i.e., translation test score) L2-proficiency measures were highly and positively correlated (r = .74, p < .001). Furthermore, the correlation analyses that we run confirmed that the L2-proficiency measure used in this experiment, even though it was self-reported, provides relevant data for the field.

[Page 32] "First, although the subjective (i.e., self-report) and objective (i.e., translation test score) L2-proficiency measures used in this experiment were positively correlated (r = .74, p < .001), future research might benefit from using standardized questionnaires to assess language proficiency [82–84]."

REVIEWER COMMENT: Discussion: Page 24, line 497: "Although this might seem obvious, in the literature on L2 false memories, until Beato and Arndt's [44] recent study, as far as we know, no one had considered this matter". Change for: "Although

this might seem obvious, in the literature on L2 false memories with the use of DRM, until Beato and Arndt's [44] recent study, as far as we know, no one had considered this matter".

RESPONSE: We have made the change that the reviewer suggested (see page 28).

REVIEWER COMMENT: Page 27, line 582: I would suggest to re-write this sentence: "As a final remark, we would like to suggest some practical implications and future research". Because what it follows it is only suggested one practical thing: "[…] future research may benefit from employing experimental procedures that study the activation processes more directly, such as, for example, the semantic priming paradigm".

RESPONSE: We rephrased this sentence and included more information in this paragraph, so the sentence that introduce the paragraph represents its content (see pages 32-33).

"We would like to highlight some limitations, suggestions for future research, and practical implications […]."

REVIEWER #2

We thank Dr. Jeffrey S. Anastasi for their thoughtful and thorough review. We believe the paper is much improved and the points clearer and more accurately stated because of his feedback.

REVIEWER COMMENT: Overall, I thought the authors did a very nice job of setting up the study, guiding the reader through the logic for the study, and explaining the need for the study. I also thought their methodology and attention to detail in setting up their study was also excellent.

> **RESPONSE:** Thank you very much for your comment. We sincerely appreciate your words.

REVIEWER COMMENT: While I think the authors did an excellent job of discussing and introducing the two theories (AMT and RHM), which I believe to be very relevant to their study, some individuals might like to see some mention of the fuzzy-trace theory. I think the AMT and RHM theories are enough, but the fuzzy-trace theory is the other primary theory in the false memory literature.

> **RESPONSE:** Thank you for the observation. As the reviewer suggests, we have mentioned the fuzzy-trace theory (FTT) on page 4.
>
> > "It is worth mentioning that these are not the only theories that have tried to explain these effects. An alternative explanation for the false memory effect is given by the fuzzy-trace theory (see [43,44] for more details). Moreover, other theoretical models that account for the second language processing are the bilingual interactive model [45] or the ontogenesis model of L2 lexical representation [46] (see [47] for discussion). In this work we have chosen to build our theoretical foundations upon the AMF and the RHM as they both make predictions in terms of activation processes. This will allow us to bridge the language and memory literature for an integrated discussion of the results."

REVIEWER COMMENT: On page 9 (in the Participants section) and page 13 (in the Procedure section), the authors mentioned that participants were only tested in either their L1 language (Spanish) or their L2 language (English). I was curious why this was done. Why not test participants in both their L1 and their L2 language?

> **RESPONSE:** Thank you for noticing that this decision was not well justified. In the Procedure section, we have included the explanation of why we did not test participants in both languages (see page 15).
>
> > "For this experiment, a blocking by language procedure [68] was used for which participants were randomly assigned to one of two language conditions: L1 (i.e., Spanish) and L2 (i.e., English). This blocking by language procedure allowed us to prevent the experimental context from promoting the intentional translation of L2 words into the L1 [69], and thus, to explore the more automatic aspects of L2 processing (see [70–72] for further discussion on the importance of language mode when studying word processing in more than one language)."

REVIEWER COMMENT: On page 14: The authors stated the participants who were tested in their L2 were asked to translate the 100 English words that they had been presented with during the study. However, on page 21 (lines 449-451) the authors mentioned that d' scores were calculated for the proportion of "yes" responses to L2 words whose meaning was known. Initially, it wasn't clear to me if the participants provided the meaning for each of the words or if they were simply asked to indicate if they knew the word meaning (a yes or no response). While I did understand what the authors were meaning here, it did take me a few different readings to fully understand what they meant. I'm not sure if this was an issue with the authors explanation of this or me being somewhat dense in not understanding it quickly. I actually would have liked to see both participants who were tested in their L1 language and those who were tested in their L2 language complete this task, so that a comparison could have been made on their general knowledge of English words. Obviously this was more important to do for those tested in their L2, so I understand why the authors did this task as they did.

> **RESPONSE:** We have included more information on page 16 (Procedure) to try to clarify how the translation test was conducted.
>
> > "Subsequently, those participants who had performed the task in their L2 carried out a translation test of all the English words that they had seen during the experiment (studied words, critical lures, distractors, and critical distractors) using a pencil and paper questionnaire. This questionnaire included a list with 100 English words and participants had to write the Spanish translation next to each word. In case they did not know or could not guess the meaning of a word, they could leave it blank and continue with the next word. Each participant's translation test response to each word was coded by a trained researcher as 1 if the translation was correct and 0 if it was incorrect."
>
> Furthermore, we agree with the reviewer in that having an assessment of the L2 word knowledge from the participants that conducted the task in their L1 would have been useful. Therefore, we have included in the Discussion section a suggestion for future research about this matter (see page 33).
>
> > "In addition, following the reviewer's suggestion, it would have been advisable to also collect the translation test data for L2 words in participants tested in their L1. This would be useful to know if both groups of participants had similar vocabulary knowledge in their L2."

REVIEWER COMMENT: A related issue is on page 15 where the authors mention that participants were most successful in knowing the meaning of the critical lures compared to any other word types (i.e., list items, distractors, etc.). I would expect that this is an important necessity for participants to recognize the critical lures and could be an alternative factor that would help explain differences in L1 and L2 lists. This could be indicative of a gist-based representation that is consistent with fuzzy-trace theory.

> **RESPONSE:** Thanks for highlighting this aspect. As the reviewer mentions, the meaning of the critical lures of L2 DRM lists was known more often than the meaning of the associates of the lists, with an almost perfect score (.985%) (see Table 3, page 18), which was vital for false memory could be observed. We have highlighted this aspect in the manuscript (see page 9, 17-18, 28). Furthermore, we believe that the 2 (word type: studied words, critical lures) x 2 (BAS: high, low) x 2 (language: L1, L2) ANOVA carried out only with the L2 known words clarifies the possible effect

that not knowing the words could have had when comparing L1 and L2 false recognition (see pages 24-25 for the results and 29 for the discussion).

REVIEWER COMMENT: On page 16 (lines 330-334): The authors mentioned that there was a Word Type x BAS interaction. However, in the comparison is seems unlikely as studied words and critical lure recognition seemed to be nearly identical for both the high-BAS (.83 and .98, respectively) and low-BAS lists (.84 and .97, respectively). Perhaps I'm missing something, but this doesn't make sense. Maybe adding a table of means for the L2 Knowledge of word meaning would be helpful to see these data more clearly. In this table the authors could provide the percent/proportion of each word type that was known by participants.

RESPONSE: Thank you for pointing out that the interpretation of this interaction was not accurate. We have changed the explanation of this interaction and, as the reviewer suggested, we have included Table 3 with the proportion of each word type that was known by participants (see pages 18-19).

"As shown in Table 3, the mean difference between high-BAS and low-BAS lists goes in the opposite direction for the studied words (i.e., the numerical value of the mean was higher for low-BAS than high-BAS lists) as compared to the critical lures (i.e., the numerical value of the mean was higher for high-BAS than low-BAS lists). However, what is crucial for the interpretation of this interaction is that the post hoc comparisons with Bonferroni correction revealed no statistically significant differences in either of these two comparisons. That is, we did not find statistically significant differences in the proportion of words whose meaning was known between high- and low-BAS lists, neither for the studied words (.83 and .84, respectively), $t(59) = -1.21$, $p = 1.000$, 95% CI [-0.04, 0.02], $d = -0.17$, nor for the critical lures (.98 and .97, respectively), $t(59) = 1.53$, $p = .773$, 95% CI [-0.01, 0.05], $d = 0.18$."

REVIEWER COMMENT: On page 19 (lines 390-392): The authors mentioned that the L2 English DRM lists created in their study produced false memories. However, it seems apparent that they did not produce false memories to the same extent as they did in the L1. This might be worth noting here.

RESPONSE: We have included a small comment on page 21 to emphasize this point.

"This confirms that the L2 (English) DRM lists created in this experiment produced the false memory effect in second-language learners, although apparently to a lesser extent than in the L1 (L2: .23 vs. L1: .45)."

REVIEWER COMMENT: On page 21 (lines 445-448): The authors mentioned that participants' who were tested in the L2 knew the meaning of fewer word presented than participants who performed the task in the L1. While this statement is probably true, I didn't think the authors tested participants for the meaning of words in the L1. I thought only L2 participants were tested on their knowledge of the meaning of words. On page 22 (lines 464-466), the authors stated that their analysis confirmed that the differences in true and false recognition between the L1 and L2 participants was not due to a lower knowledge of L2-word meaning. Again, if only the L2 participants were tested on their knowledge of the word meanings, this seems somewhat confusing.

RESPONSE: In our view, as in previous DRM studies, it is safe to assume that undergraduate students know the meaning of the L1 words included in the experiment (e.g., balón, cama, sonrisa, invierno, etc., meaning ball, bed, smile, and winter, respectively). However, the reviewer is right in that participants were not tested for their knowledge of L1 words in our experiment. We have added some information on page 24 (lines 495-497) and rephrase a sentence on page 25 (line 521) to make it clearer that we only assessed knowledge of L2-word meaning.

REVIEWER COMMENT: I thought the authors' explanation for why participants showed more false memories for the L2 word lists was higher with the high-BAS lists than the low-BAS lists on page 25 was very clear and organized. It appears that they authors found a nice pattern of results such that false memory was highest in L1 for high-BAS lists, moderate in L1 for low-BAS lists and also moderate for L2 high-BAS lists, and lowest for L2 for low-BAS lists. These seem to indicate the importance of critical lure activation that would be expected for the AMT. The only shortcoming that I see is that I don't think the authors made the distinction between the cognitive distinction between the L1/low-BAS lists and the L2/high-BAS lists. What do the authors think is going on between these two conditions and how might they be explained with the AMT, RHM, or Fuzzy-trace theories?

RESPONSE: Thank you for your comment. We believe that it is indeed very interesting and worth highlighting this idea. In the Results section (see pages 26-27) we have compared the false recognition between L1/low-BAS lists and L2/high-BAS lists. Furthermore, in the Discussion section we have also provided an explanation for this result (see pages 31-32).

[Pages 26-27] "Second, looking into the overall pattern of false recognition, the highest false recognition rate was obtained in the L1 for high-BAS lists, followed by a moderate false recognition rate in the L1 for low-BAS list, but also in the L2 for high-BAS lists. In fact, the false recognition did not differ between L1 low-BAS lists and L2 high-BAS lists, $t(59) = -1.00$, $p = 1.000$, 95% CI [-0.67, 0.30], $d = -0.18$. Lastly, the lowest false recognition rate was obtained in the L2 for low-BAS list."

[Pages 31-32] "Looking at the overall pattern of levels of activation throughout the L1 and L2 (see Fig. 1), we reported an interesting pattern of results. The L1 critical lures form high-BAS lists were the ones that received the highest activation in our experiment, followed by the moderate activation received by the L1 critical lures from low-BAS lists and the L2 critical lures from high-BAS lists, which did not differ between them. Finally, the L2 critical lures from low-BAS lists, as it was previously mentioned, barely received any activation. This seems to indicate the importance of critical lure activation that would be expected for the AMF. This pattern of results could be explained by the ontogenesis model of L2 lexical representation [46,55] as it states that semantic-level representations of L2 words, as compared to L1 words, have fewer connections and, especially important for our data, these connections tend to be weaker. With this in mind, we can infer from our results that strongly associated words in the L2 would be associated in second-language learners with a similar level of associative strength as weakly associated words in their L1."

REVIEWER COMMENT: Overall, I enjoyed the article and thought the authors did an excellent job at conducting, presenting, and explaining their research. Below I have a few small wording changes to help the authors. Wording Suggestions

Line 44: change wording – change from "has been favored by the fact" to "has been influenced by the fact"

Line 83: change wording – change from "the critical lures has" to "the critical lures have"

Line 161: change wording – change "cero" to "zero"

Line 168: change wording – change "Arndt" to "Arndt's"

Line 190: change wording – change "when they had" to "when they were"

Line 365: change wording – change "Once we have proofed" to "Once we have proved"

Line 515: change wording – change "cero" to "zero"

> **RESPONSE:** Thank you very much for your suggestions. They have certainly improved the article. We have included all the wording suggestions in the revised manuscript.

REVIEWER #3

We thank Dr. Kristen Diliberto-Macaluso for feedback on the manuscript. The manuscript is stronger and clearer because of her comments.

REVIEWER COMMENT: 1. One question I had was why the authors' chose to use different DRM lists for their high and low BAS conditions? In Beato and Arndt's work, they kept the critical lure constant, using top four and bottom four associates of the same critical lure to create the high and low BAS conditions, respectively. This was done to ensure that there were any item differences that could occur with using different lists. Though BAS values did not differ across DRM list language, can the authors be sure there are no item differences? In addition, why did the authors manipulate DRM list language as a between-subject variable rather than a within-subject variable?

> **RESPONSE:** We thank the reviewer for bringing up this matter. Regarding the first question, it is true that Beato and Arndt tried to control the materials, the DRM lists, as much as possible, and so, they built two DRM lists for each critical lure, a high-BAS list and a low-BAS list. The authors believed that this method of list construction ensured that the effect of BAS on critical lure false memory were not due to item differences that could occur if high- and low-BAS lists were associated with different critical lures, also hoping to find similar knowledge of L2-word meaning between high- and low-BAS lists. Unfortunately, the results obtained in this study confirmed that constructing the lists in this way could lead to differences in the knowledge of L2-word meaning between high-BAS and low-BAS lists.
>
> When building our Spanish and English DRM lists in the present study, we took into account this evidence trying to further control the characteristics of the lists. Specifically, the Spanish and English DRM lists were selected so that the high-BAS lists had significantly higher BAS values than the low-BAS lists, and they did not differ in terms of FAS values, being these values extremely low (virtually zero) for all lists. Furthermore, Spanish and English DRM lists did not differ in their BAS values, nor in their FAS values. Additionally, after conducted a previous normative study, we only

included words whose meaning was widely known, on average, by 85.56%. And finally, we confirmed that high- and low-BAS lists did not differ in the estimated knowledge of word meaning. All these controls, and the limited number of words from the Spanish and English databases used, made it literally impossible to build a high and a low-BAS list for each critical lure, but instead we were able to address the issue highlighted by Beato & Arndt. These are the reasons why we used different critical lures for our high and low-BAS lists.

Regarding the second question, in the Procedure section we have included information about why language was manipulated as a between-subject variable rather than within-subject variable (see page 15).

> "For this experiment, a blocking by language procedure [68] was used for which participants were randomly assigned to one of two language conditions: L1 (i.e., Spanish) and L2 (i.e., English). This blocking by language procedure allowed us to prevent the experimental context from promoting the intentional translation of L2 words into the L1 [69], and thus, to explore the more automatic aspects of L2 processing (see [70–72] for further discussion on the importance of language mode when studying word processing in more than one language)."

REVIEWER COMMENT: 2. Another question I had is about fluency. Though the authors report that participants self-reported as moderately fluent (M = 6.00) and that there were no differences between participants across each list language group, the authors do not mention predictions made by the revised hierarchical model with respect to fluency. In their E2, Beato and Arndt find that the effects of BAS on false recognition in L2 are also influenced by differences in participants' fluency. It is expected that as participants become more fluent in their L2 that the links between L2 and concepts strengthen, which also has implications for activation monitoring framework. Thus, it appears that irrespective of L2 word knowledge, fluency may play an important role in the reported BAS effects on false recognition in L2.

RESPONSE: Thank you for raising this important topic. In the Introduction we have mentioned the predictions of the RHM with respect to proficiency (see pages 5-6). Among the goals of this work was not to compare different levels of proficiency, as Beato & Arndt did. Nevertheless, it would be interesting to include this aspect in future studies, for what we have included this as a future research line in the Discussion (see page 33).

> [Page 5-6] "Nonetheless, once L2 learners become more proficient, the conceptual links from L2 words to the concepts become stronger."

> [Page 33] "Second, it would be interesting to examine whether L2 proficiency moderates the results reported in the present experiment using participants with a wider range of L2 proficiency."

For a further discussion in L2 proficiency, please, see Editor comment 4 in which we explained some analyses that we run to examine whether L2 proficiency moderates our results (see pages 25-26 in the Results section and page 30 in the Discussion section).

REVIEWER COMMENT: 3. I guess I am also curious about cross-language effects (L1 studied list to L2 test, vice versa), as I think it would really test the revised hierarchical model and AMF as well as the effects of BAS.

> **RESPONSE:** We have considered how interesting it would be to carry out research in cross-language effect to test these theories. To address this point, in the Discussion section we have included the following information (see page 33).
>
> > "Fourth, cross-language effects should be also addressed in future studies to further assess the theoretical frameworks (revised hierarchical model and activation monitoring framework)."

REVIEWER COMMENT: 4. On page 19, the authors perform a repeated measure ANOVA and indicate they used a Bonferroni correction for post hoc tests. The authors report a significant pairwise comparison finding a higher proportion "yes" responses to critical lures than to distractors. The exact p-value reported is .038. Is this value significant when applying the Bonferroni correction for experimentwise error? How many tests are being performed here?

> **RESPONSE:** The *p*-value reported (.038) is the *p*-value already adjusted for comparing a family of 6 estimates (calculated using JASP). The 6 comparisons were: 1. Studied vs. Critical lures; 2. Studied vs. Distractors; 3. Studied vs. Critical distractors; 4. Critical lures vs. Distractors; 5. Critical lures vs. Critical distractors; and 6. Distractors vs. Critical distractors. The non-adjusted *p*-value for the comparison "Critical lures vs. Distractors" would be $p = .003$, which is statistically significant when computing 6 comparisons and applying Bonferroni correction as the new alpha would be set at .008. JASP already adjusted the .003 *p*-value for comparing a family of 6 estimates using Bonferroni and the result was a *p*-value of .038. This allows us to compare the adjusted *p*-value (.038) to the .050 threshold. Thank you for noticing that this was not clear in our manuscript. In order to clarify this issue, we included the following information in the Data analysis section (see page 17).
>
> > "Bonferroni correction was used for post hoc tests and the p-value reported in these tests were the adjusted values for multiple comparisons."

REVIEWER COMMENT: 5. Similarly, on page 22, the authors report a "marginally significant interaction with Word type x BAS). Given the reported p-value is .053, this value is not significant if using a .05 significance value. In my opinion, the authors should not have proceeded with conducting post-hoc analyses on their data.

> **RESPONSE:** Thank you for your comment. In order to address this issue, and following the editor's suggestion, we have better justified the post hoc analyses that we conducted after this marginally significant interaction by stating our a priori hypothesis (see page 25).
>
> > "Finally, there was a marginally significant interaction Word type x BAS, $F(1, 118) = 3.83$, $p = .053$, $\eta^2_p = 0.031$. As we had an a priori hypothesis regarding this interaction (i.e., BAS will have an effect in false recognition, but not in true recognition), we continue with the analysis of this marginally significant interaction."

REVIEWER COMMENT: Minor typo. Reference #57, Diliberto(-Macaluso) is misspelled as Deliberto.

RESPONSE: Sorry for the typo. That reference is now correct (see reference #63).
