# Supplementary material for: False memory in a second language: The importance of controlling the knowledge of word meaning
Source: PLoS One. 2023 May 11;18(5):e0285747. doi: 10.1371/journal.pone.0285747 (PMC10174556; doi:10.1371/journal.pone.0285747)
Supplement: Attachment — Submitted filename: Response to Reviewers.pdf [file pone.0285747.s002.pdf]

Response to Reviewers

[PONE-D-22-27134]

JOURNAL REQUIREMENTS:

Please review your reference list to ensure that it is complete and correct. If you have cited papers that have been retracted, please include the rationale for doing so in the manuscript text, or remove these references and replace them with relevant current references. Any changes to the reference list should be mentioned in the rebuttal letter that accompanies your revised manuscript. If you need to cite a retracted article, indicate the article's retracted status in the References list and also include a citation and full reference for the retraction notice.

> **RESPONSE:** The authors believe that the reference list is complete and correct, and that they have not cited any retracted paper. We corrected a typo in the journal name of reference 59, and included three new references that were needed to properly implement the changes suggested by reviewer 4. The new references are the followings:
>
> 49. Beato MS, Suarez M, Cadavid S. Disentangling the effects of backward/forward associative strength and theme identifiability in false memory. Psicothema. 2023;35: 178–188. doi:10.7334/psicothema2022.288
> 69. Duchon A, Perea M, Sebastián-Gallés N, Martí A, Carreiras M. EsPal: one-stop shopping for Spanish word properties. Behav Res Methods. 2013;45: 1246–1258. doi:10.3758/s13428-013-0326-1
> 70. Brysbaert M, New B. Moving beyond Kučera and Francis: a critical evaluation of current word frequency norms and the introduction of a new and improved word frequency measure for American English. Behav Res Methods. 2009;41: 977–990. doi:10.3758/BRM.41.4.977

REVIEWER #4

REVIEWER COMMENT: I have received the revision of this work and reviewed all previous reviewers' comments and the authors' responses. I do recommend the publication of this version. I find that they have successfully improved the former manuscript following the recommendations of the reviewers and the editor. I enjoyed reading this work, and I believe it is well-motivated, with a test of important theoretical approaches in both the field of false memory and bilingualism. It is well-written, and the methods are rigorous, with exquisite control of the materials used. Also, I appreciate that they made available materials and data.

RESPONSE: We thank Reviewer 4 for the kind words and the feedback on our manuscript. All the thoughtful comments on our submission have been considered and implemented. We believe that the manuscript is stronger and clearer now.

REVIEWER COMMENT: Page 7, line 145. In the introduction, I recommend succinctly explaining, with a couple of words, some important concepts for potential readers that might not be familiar with them. For example, this research might interest psycholinguists unfamiliar with BAS and FAS or the main idea of the fuzzy-trace theory.

RESPONSE: Thank you for raising this important topic. As suggested, we have explained, with a couple of words, the concepts of BAS and FAS, and the main idea of the fuzzy-trace theory. We have also referred the reader to useful literature about these topics. We think that having included these brief explanations will be useful to some readers. The text included in the manuscript is as follows:

[Page 6/lines 130-131] "Briefly, BAS refers to the associative strength from the studied items to the critical lure [21,22,49]."

[Page 7/lines 148-150] "Finally, Beato and Arndt used DRM lists that varied in the levels of both backward (BAS) and forward associative strength (FAS or the associative strength from the critical lure to the studied items; see [49] for a recent study independently examining the effect of BAS and FAS on false memory)."

[Page 4/lines 77-79] "An alternative explanation for the false memory effect is given by the fuzzy-trace theory, which states that false memory appears when the gist information of the list (matching the critical lure) is extracted, and the retrieval of verbatim representations is not enough to reject the critical lure (see [43,44] for more details)."

REVIEWER COMMENT: In the methods, did the authors control for frequency in the lists/critical lures? Word frequency is important when dealing with concept retrieval in activation accounts. As a reader, I would like to know whether it has impacted the BAS manipulation (i.e., the high-BAS words could potentially present greater frequency than low-BAS and differences within each language).

RESPONSE: We thank the reviewer for bringing this matter up. Yes, we control for frequency (and word length) in high-BAS vs. low-BAS lists in both languages. We agree with the reviewer that frequency is an important variable when dealing with lexical access and concept retrieval. We have made that information available for the reader in the method section:

[Page 13/lines 264-268] "Furthermore, words from the two BAS conditions did not differ in terms of word length (i.e., number of letters), $t(14) = 1.78$, $p = .097$, 95% CI [-0.10, 1.06], $d = 0.89$, nor in their frequency (i.e., logarithmic frequency from EsPal [69]), $t(14) = 0.62$, $p = .542$, 95% CI [-0.22, 0.40], $d = 0.31$, which was, on average, 1.13 ($SD = 0.30$) for high-BAS lists and 1.22 ($SD = 0.27$) for low-BAS lists."

[Page 14/lines 291-294] "At the same time, high- and low-BAS L2 lists did not differ in their FAS values, $t(14) = -0.85$, $p = .409$, 95% CI [-0.01, 0.01], $d = -0.43$, word length, $t(14) = 0.29$, $p = .779$, 95% CI [-0.59, 0.77], $d = 0.14$, or frequency (i.e., logarithmic frequency based on

SUBTLEX$_{US}$ [70]), $t(14) = 0.48$, $p = .641$, 95% CI [-0.19, 0.30], $d = 0.24$, with frequency mean values of 1.61 ($SD = 0.22$) and 1.66 ($SD = 0.24$) for high- and low-BAS lists, respectively."

REVIEWER COMMENT: Finally, a couple of typos:
Page 28, line 627. In "These results are consistent with previous studied", it should be "studies".
Page 8, line 166. "the BAS effect occur" should be "occurs".

RESPONSE: Thank you very much for your suggestions. We have corrected the typos in the revised manuscript. The new text now appears in page 8 (lines 169-170) and page 30 (lines 638).
